# Single-cell RNA sequencing reveals TCR^+^ macrophages in HPV-related head and neck squamous cell carcinoma

**DOI:** 10.3389/fimmu.2022.1030222

**Published:** 2022-10-27

**Authors:** Yourong Jiang, Siwei Zhang, Lu Tang, Rui Li, Jinglei Zhai, Suisui Luo, Yiman Peng, Xiaohang Chen, Lanlan Wei

**Affiliations:** ^1^ The First Affiliated Hospital of Harbin Medical University, School of Stomatology, Harbin Medical University, Harbin, Heilongjiang, China; ^2^ Department of Microbiology, Harbin Medical University, Harbin, Heilongjiang, China; ^3^ Wu Lien-Teh Institute, Harbin Medical University, Harbin, Heilongjiang, China; ^4^ School of Medicine, Southern University of Science and Technology, Shenzhen, Guangdong, China; ^5^ Institute for Hepatology, The Third People’s Hospital of Shenzhen, Shenzhen, Guangdong, China; ^6^ The Genetics Laboratory, Longgang District Maternity & Child Healthcare Hospital of Shenzhen City, Shenzhen, Guangdong, China

**Keywords:** human papillomavirus, head and neck squamous cell carcinoma, macrophages, single-cell sequencing, tumor microenvironment

## Abstract

The prognosis of human papillomavirus (HPV)-infected head and neck squamous cell carcinoma (HNSCC) is often better than that of HPV^-^ cancer, which is possibly caused by the differences in their immune microenvironments. The contribution of macrophage, as a principal innate immune cell, to this phenomenon is still unclear. In this study, a single-cell atlas of 4,388 high-quality macrophages from 18 HPV^-^ and 8 HPV^+^ HNSCC patients was constructed with single-cell RNA sequencing data. Eight macrophage subsets were identified from HNSCC, whereas their functional properties and developmental trajectory were delineated based on HPV status. Our results demonstrated that macrophages in HPV^+^ HNSCC exhibit stronger phagocytic ability, although the infiltration rate of macrophages decreased. From the results, a unique macrophage subset with TCR and CD3-specific signatures was identified from HPV-related HNSCC. These TCR^+^ macrophages potentially participate in the regulation of the TCR signaling pathway and phagocytosis. In conclusion, our results suggested that HPV could affect the infiltration rate, function, and differentiation of macrophages in HNSCC, whereas TCR^+^ macrophages play a critical role in the HNSCC microenvironment. These results provide new insights into the immune microenvironment of HNSCC and offer a valuable resource for the understanding of the immune landscape of HPV-related HNSCC, which will in turn help the development of immunotherapy strategies for the disease.

## Introduction

Head and neck squamous cell carcinoma is the sixth most common cancer worldwide with an annual incidence of 650,000 cases and arises from the mucosal epithelium in the oral cavity, oropharynx, nasopharynx, and larynx (1, 2). The risk factors of HNSCC include smoking and alcohol consumption, whereas human papillomavirus has emerged as a novel pathogenic factor (1, 2). Clinically, the prognosis of HPV^+^ HNSCC patients is better, probably because of the impact of HPV on the immune cells in the tumor microenvironment (TME) (3). However, the mechanism underlying this difference in prognosis remains unclear.

Macrophages represent a major constituent of immune cells in the TME and are able to stimulate key steps in tumor progression. They usually exhibit remarkable plasticity after being recruited into the TME and are skewed away from the tumoricidal phenotype (M1) toward a tumor-promoting phenotype (M2) (4, 5). For HNSCC, most infiltrated macrophages are M2-like tumor-associated macrophages (TAMs) which are correlated with tumor metastasis and poor prognosis (6, 7). Compared with HPV^-^ HNSCC, a higher M1/M2 ratio of infiltrating macrophages in HPV^+^HNSCC may be associated with its favorable prognosis (8). However, the specific function of macrophages in HNSCC has not been fully described. It is necessary to systematically characterize macrophages within the HNSCC microenvironment.

Recent developments in single-cell RNA sequencing (scRNA-seq) have enabled the classification of macrophages. In recent years, a unique subset of macrophages, TCR^+^ macrophages, has been discovered to exist in substantial tumors and diseases (9). TCR^+^ macrophages have been reported to be present in tuberculous granulomas, atherosclerosis (10), and several types of cancer (colon cancer, esophageal cancer, hepatic carcinoma, and melanoma) (10, 11); these unique cells could enhance phagocytosis and secrete IFN-γ, TNF, MIP-1β, and CCL2 to promote inflammation (11–13). However, the presence and functions of these cells in HPV-related HNSCC are still unknown.

Here, we analyzed and characterized macrophages at single-cell resolution using a systematic approach. To the best of our knowledge, this is the first study to provide a comprehensive analysis of macrophage subsets and the effect of HPV infection on the regulation of macrophage subsets in HNSCC. Moreover, our analyses demonstrate the presence of TCR^+^ macrophages which are involved in the TCR signaling pathway and exhibit a stronger phagocytic ability in the HNSCC TME for the first time. Altogether, these analyses provide new insights into the immune microenvironment of HNSCC and could serve as a valuable theoretical basis for future studies on the immune regulation of HPV-related HNSCC.

## Materials and methods

### Patient characteristics

HNSCC samples were collected after tumor resection surgery of patients with head and neck squamous cell carcinoma at the Cancer Hospital Chinese Academy of Medical Sciences, Shenzhen Center. All participants provided written informed consent regarding this study, and ethical approval for the study was obtained from the Ethics Committee of The Third People’s Hospital of Shenzhen [2021-057].

### Data collection

The scRNA-seq data of 18 HPV^-^ HNSCC and eight HPV^+^ HNSCC patients used in this article were obtained from the GEO database (https://www.ncbi.nlm.nih.gov/geo/), and the accession number was GSE139324. RNA-seq data and clinical information of 520 HNSCC patients were obtained from TCGA database (https://www.cancer.gov/).

### Quality control and RNA-seq data processing

After downloading scRNA-seq data from the GEO dataset, we first processed the data with Seurat 3.0 in R (version 4.1.2) to remove low-quality cells. Cells would be flagged as poor-quality ones if they met one of the following thresholds: 1) the number of expressed genes lower than 500 or larger than 3,500; 2) 15% or more of UMIs were mapped to the mitochondria; 3) 0.1% or more of UMIs were mapped to hemoglobin genes; and 4) 3% or more of UMIs were mapped to ribosomal genes. Mitochondrial, ribosomal, and hemoglobin genes were filtered to avoid interference with subsequent analysis. A total of 20,326 genes in a total of 58,656 cells were detected. Then, we utilized functions in the Seurat package to normalize and scale the single-cell gene expression data. Then, principal component analysis (PCA) was performed according to the standard analysis process and identified 28 significant principal components. Cell clustering analysis was carried out with the parameter resolution of 1.2 and 31 Seurat clusters found. These clusters were annotated with marker genes from CellMarker (http://biobigdata.hrbmu.edu.cn/CellMarker/) (14).

Macrophages and monocyte-derived macrophages were extracted while dimension reduction was performed using PCA with 19 significant principal components. These cells were then clustered with the parameter resolution of 0.6, and 11 clusters were identified. Among these 11 clusters, three of them were removed from subsequent analysis as one cluster was identified as epithelial cells and two clusters contained too few cells to be analyzed.

### Pseudotime estimation

Pseudotime trajectory analysis was constructed based on Monocle2 (version 2.18.0, R package) following the tutorial to order the macrophage subsets in HPV^+/-^ HNSCC. In order to avoid omitting some important genes, we selected all genes for completely unsupervised trajectory analysis. For differentiation trajectory analysis, top 50 pseudotime-related differentially expressed genes were selected and divided into three clusters for further analysis.

### Cell communication analysis

Cell–cell communication atlases between different cell subsets were explored with CellChat (Version 1.1.3, R package) following the standard protocol (15).

### Functional enrichment analysis

The top 50 genes (or all genes in the case of less than 50) with either significant upregulation or downregulation were selected based on results of differential gene expression analysis. Functional enrichment of selected genes was performed using DAVID 6.8 (https://david.ncifcrf.gov/) (16). A pathway enrichment analysis of different immune cells was performed with irGSEA (R package, https://github.com/chuiqin/irGSEA/). Phagocytosis and antigen presentation function of macrophage subclusters were analyzed by GSVA (R package,1.38.2). All genes associated with antigen presentation and phagocytosis available in Molecular Signatures Database (MSigDB, v7.5.1) were collected and used as reference genes for pathway enrichment analysis.

### Immune infiltration evaluation and prognostic analysis

Immune infiltration of eight macrophage subsets was analyzed by CIBERSORT. We categorized the samples into high or low infiltration groups based on the mean infiltration rates of immune cells of the patients. Survfit (version 3.3-1, R package) and Survdiff (version 0.4.9, R package) were used to generate Kaplan–Meier survival curves and calculate the *p*-value of the log-rank test. Survival analysis of total macrophage infiltration in HNSCC was calculated by Timer 2.0 (https://cistrome.org) (17).

### Immunofluorescence staining

Immunofluorescence staining was performed as previously reported (18). Briefly, paraffin-embedded (FFPE) samples were sectioned at 3-µm thickness. A 0.01-M citrate buffer solution (pH 6.0) was used for retrieval treatment for 15 min in a pressure cooker. After blocking with 5% BSA at room temperature for 30 min, a mixture of three different primary antibodies, namely, CD3 (1:200 dilution, Cat No.17617-1-AP, Proteintech), CD68 (1:100 dilution, Cat No. 66231-2-lg, Proteintech), and TCR α (1:100 dilution, Abcam, ab18861), was added and incubated overnight at 4°C. Then, slides were incubated with the mixture of Coralite488-conjugated anti-mouse IgG (H + L) secondary antibody (1:250 dilution, Cat No. SA00013-1, Proteintech) and Coralite594-conjugated anti-rabbit IgG (H + L) secondary antibody (1:250 dilution, Cat No. SA00013-4, Proteintech) at 37°C for 30 min. A fluorescent quenching sealing agent with DAPI was dropped on the slides and covered with slip carefully. Images were taken using a Leica TCS Sp8 laser scanning confocal microscope at the appropriate excitation wavelength for the fluoroscope.

### Statistical analysis

The statistical significance for differential gene analysis of scRNA-seq data was calculated by Wilcox test using the Seurat R package. The statistical significance in the proportions of different infiltrated immune cells and the infiltration rate of macrophage subsets among the immune cells in HPV^+/-^ HNSCC samples were assessed using the Mann–Whitney test in GraphPad Prism 6 software. Statistical significance was determined at *p* < 0.05. All experiments were repeated three times and at least three samples per experiment.

## Results

### A single-cell atlas of CD45^+^ immune cells in HPV^+/-^ HNSCC

**Figure 1:**
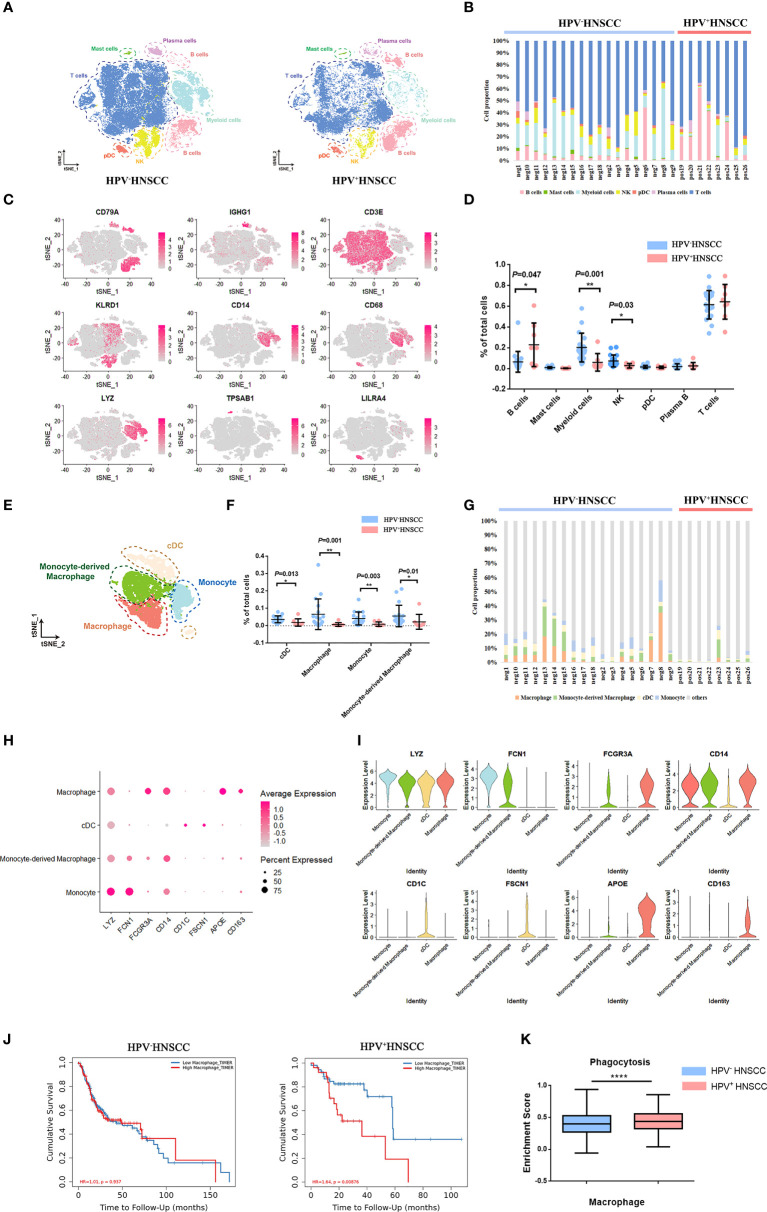
Single-cell atlas of HPV-related HNSCC. **(A)** The t-SNE plot of high-quality cells in HPV^-^ (left) and HPV^+^ (right) HNSCC. pDC, plasmacytoid dendritic cell. NK, natural killer cell. **(B)** The proportion of immune cells in each sample. **(C)**The feature plot of the feature gene distribution. **(D)** The frequency of immune cell clusters. HPV^+^ HNSCC (n = 8), HPV^-^ HNSCC (n = 18). **(E)** The t-SNE plot of myeloid cells in HPV-related HNSCC. cDC, classical dendritic cell. **(F)** The proportion of myeloid cell subsets. **(G)** The frequency of macrophages and monocyte-derived macrophages in each sample. **(H, I)** The bubble charts and violin plot of classical markers indicating group identities. **(J)** Prognosis analysis of HPV^+/-^ HNSCC patients from TCGA datasets calculated by TIMER2. HPV^+^ HNSCC (n = 98), HPV^-^ HNSCC (n =422). **(K)** The expression of phagocytosis signatures in macrophage across HPV^+/-^ HNSCC patients. *P < 0.05, **P < 0.01, ****P < 0.0001.

To explore the characteristics of macrophages in HPV^+/-^ HNSCC, scRNA-seq data of tumor-infiltrating immune cells from 18 HPV^-^ and 8 HPV^+^ HNSCC patients were obtained from the GEO dataset (GSE139324) with a total of 58,656 quality control compliant cells. Dimension reduction and unsupervised cell clustering were performed using the Seurat R package. B cells (CD79A), plasma cells (CD79A and IGHG1), T cells (CD3E), NK cells (KLRD1), myeloid cells (CD14, CD68, and LYZ), mast cells (TPSAB1), and plasmacytoid dendritic cells (LILRA4) were firstly identified from the t-distributed stochastic neighbor embedding (t-SNE) result (**Figure 1A**), and the representative characteristic gene expressions of each cluster were shown (**Figure 1C**; **Supplementary Figure 1A**). The proportion of infiltration of different cell clusters in each patient is illustrated in **Figure 1B**. As compared with HPV^-^ HNSCC patients, the proportion of B cells increased while the proportions of myeloid cells and NK cells decreased in HPV^+^ HNSCC (**Figures 1B, D**). Based on the expression of the feature markers, myeloid cells were further classified into macrophages (APOE, CD163, and FCGR3A), monocyte-derived macrophages (FCN1 and FCGR3A), classical dendritic cells (CD1C and FSCN1), and monocytes (FCN1) (**Figure 1E**). The feature markers of these clusters are shown in **Figures 1H, I**. The proportion of these four clusters in each patient was calculated. The results showed that the infiltration rate of macrophages and monocyte-derived macrophages increased significantly in HPV^-^ HNSCC (**Figures 1F, G**). As calculated by Timer 2.0, lower macrophage infiltration in HPV^+^ HNSCC was positively correlated with the better prognosis of 98 patients from TCGA dataset (**Figure 1J**). Moreover, the phagocytosis score of macrophages was significantly increased in HPV^+^ HNSCC (**Figure 1K**). These results suggested that HPV infection may affect the infiltration level and function of macrophages in HNSCC.

### Characterization of the single-cell expression profiles for macrophage cell lineages in HPV^+/-^ HNSCC

**Figure 2:**
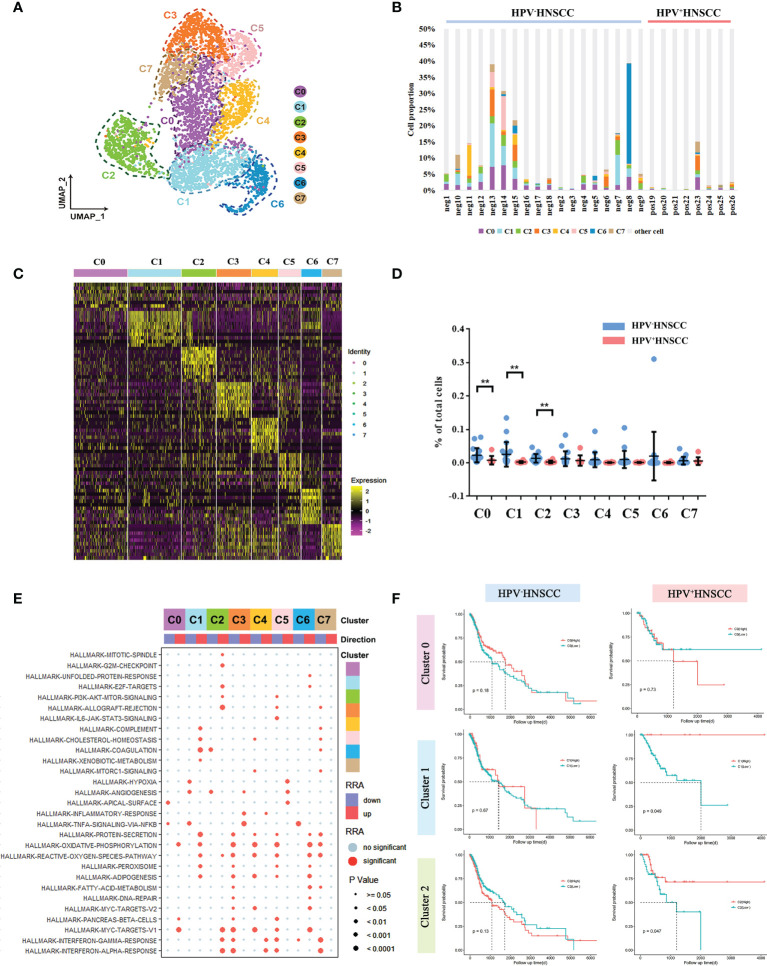
Macrophage subtype analysis based on single-cell gene expression. **(A)** The UMAP plot of macrophage subsets in HPV-related HNSCC. **(B)** The proportion of macrophage subsets in each sample. **(C)** The heatmap of the top 10 genes differentially expressed in each macrophage subset. **(D)** The frequency of macrophage subsets in total cells with HPV^+/-^ HNSCC scRNA datasets **P < 0.01. **(E)** The bubble chart of statistically significant differences in specific gene sets from MSigDB with each macrophage subgroup. Light blue points represent no significant difference. The cluster tree on the left represents the similarity of expression patterns of different gene sets in different macrophage subsets. **(F)** Prognostic significance of C0, C1, and C2 assessed in HPV^+/-^ HNSCC.

Based on the scRNA-seq data, we categorized the macrophages into eight subclusters and observed a high heterogeneity among the clusters (**Figure 2A**). The gene expression and enriched pathways of these eight macrophage subclusters were also different (**Figures 2C, E**). A functional enrichment of the significantly upregulated genes in these eight subclusters was analyzed and is listed in **Supplementary Table 1**. In addition, the significantly enriched biological processes in macrophage subsets were compared between HPV^+/-^ HNSCC. The results showed that macrophages in HPV^+^ HNSCC were involved in DNA damage repair pathways (**Supplementary Figures 1C, D**), which is consistent with our previous findings (19).

Next, we compared the levels of infiltration of macrophage subsets between HPV^+/-^ HNSCC patients and found that infiltration rates of C0, C1, and C2 cells were lower in HPV^+^ HNSCC (**Figures 2B, D**), whereas there was no significant difference in that of the other subsets. Then, the effect of HPV on the gene expression of these three groups was analyzed, respectively. The functional enrichment of the highly differentially expressed genes in HPV^+^ HNSCC patients were analyzed, and the results showed that the macrophage-related immune response was more active (**Supplementary Figure 1B**). Remarkably, the antigen presentation and phagocytosis of macrophage-related genes, HLA-DQA2 and IGLC2, were highly expressed in all these three macrophage subsets in HPV^+^ HNSCC patients (**Supplementary Figure 2**). Moreover, the patients’ survival rates assessed using TCGA data showed that the prognosis of patients with higher C1 and C2 infiltrations was better (**Figure 2F**), whereas there was no significant difference in that of HPV^-^ HNSCC patients. These results suggested that HPV could simultaneously affect the infiltration level and function of macrophage subsets and subsequently alter the prognosis of HNSCC.

### Pseudotime state transition of macrophages in HPV^+/-^ HNSCC

**Figure 3:**
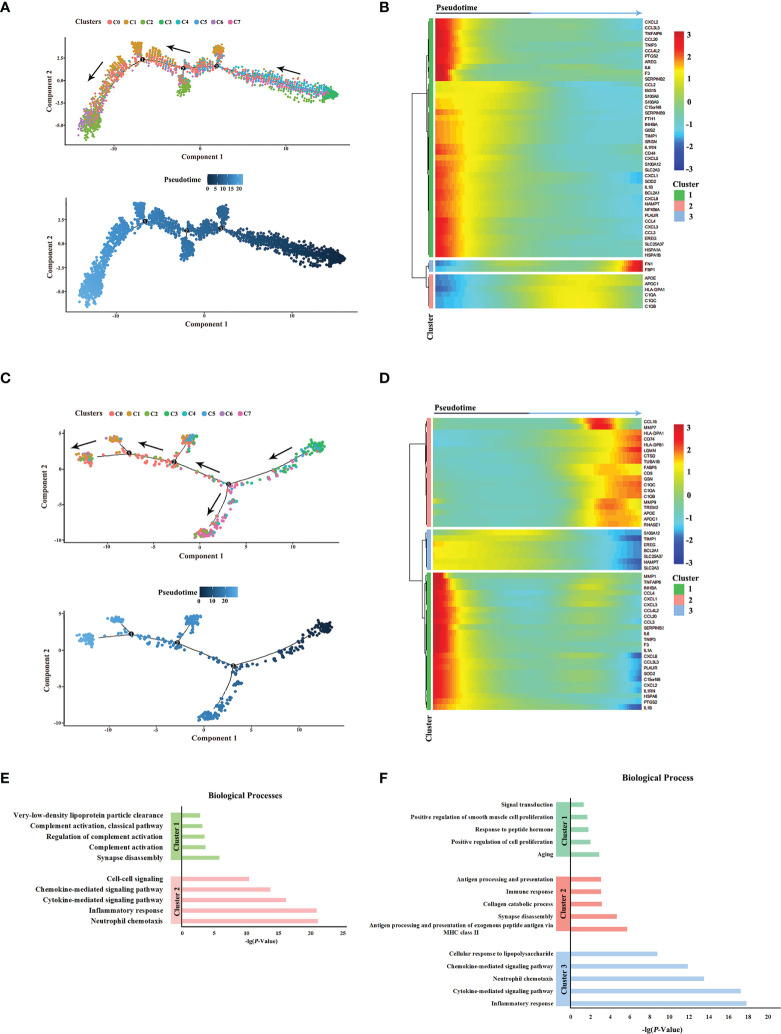
Pseudotime analysis of macrophage subsets in HPV^+/-^ HNSCC. **(A)** Simulated differentiation trajectory analysis of macrophage subsets in HPV^-^ HNSCC. Each point corresponds to a single cell, and each color represents a macrophage cell cluster. **(B)** The heatmap of the expression of the top 50 genes with the pseudotime value in HPV^-^ HNSCC. The genes with a similar expression trend converge to form different clusters. **(C)** Simulated differentiation trajectory analysis of macrophage subsets in HPV^+^ HNSCC. **(D)** The heatmap of the expression of the top 50 genes with the pseudotime value in HPV^+^ HNSCC. **(E)** Functional enrichment analysis of different clusters in HPV^-^ HNSCC. **(F)** Functional enrichment analysis of different clusters in HPV^+^ HNSCC.

Next, the differentiation trajectories of macrophages were analyzed under different HPV statuses by pseudo-temporal analysis in order to determine whether HPV would affect the differentiation of macrophages. The results showed that the trajectories of macrophage cell subsets in HPV^+/-^ HNSCC were roughly the same (**Figures 3A, C**). In addition, the differential gene expression along trajectories were analyzed and the top 50 genes with the most significant difference were selected for downstream analysis. These genes can be clustered into three groups, according to their changes in the trajectories. The composition of the genes in these three clusters between HPV^+/-^ HNSCC varied slightly, suggesting that the hub genes involved in cell differentiation were different between HPV^+/-^ HNSCC (**Figures 3B, D**). The functional enrichment analysis of these genes was carried out, and the results showed that the antigen presentation ability of macrophages significantly increased in HPV^+^ HNSCC (**Figures 3E, F**). These results showed that HPV had functional and quantitative effects on infiltrated macrophages around HNSCC.

### Identification and confirmation of TCR^+^ macrophages in HPV^+/-^ HNSCC

**Figure 4:**
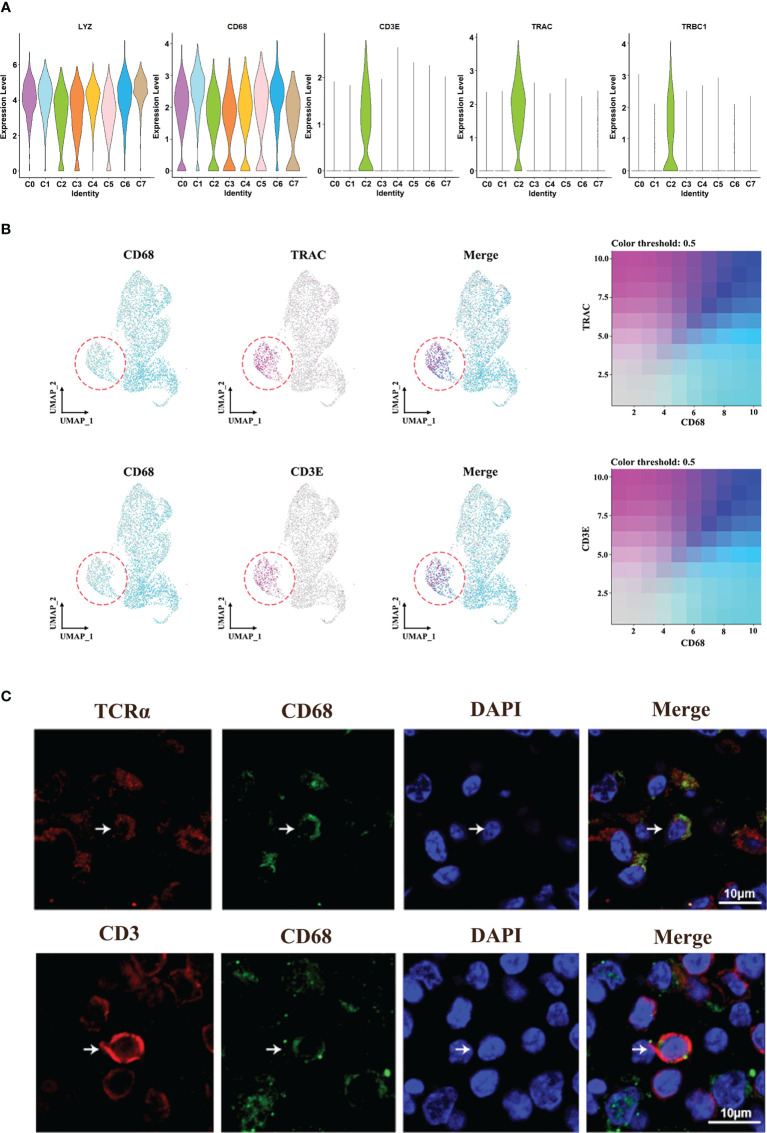
Identification of TCR^+^ macrophages. **(A)** Violin plot of the specific marker genes in cluster 2. **(B)** UMAP plot of cells with gene co-expression cells in cluster 2. **(C)** Representative immunofluorescence diagram of CD3^+^ CD68^+^ macrophage and TCR^+^CD68^+^ macrophage in HNSCCs.

Interestingly, we found a C2-specific subset of macrophages expressing TCR marker genes (TRAC and TRBC1) and CD3-specific signatures (CD3E) (**Figures 4A, B**). Totally, TCR^+^ macrophages account for 0% to 46% of all macrophages across patients (**Supplementary Figure 3**). In addition, immunofluorescence analysis of HNSCC samples showed the co-localization of CD68 and CD3/TCRα on the cell membrane, respectively (**Figure 4C**; **Supplementary Figure 3A**), and these cells were usually isolated in areas with high concentrations of T cells, suggesting the association between this group of cells and T cells.

In order to verify the characteristics of TCR^+^ macrophages, the single-cell sequencing data of T cells and macrophages were analyzed. The results showed that the characteristics of TCR^+^ macrophages were more consistent with the characteristics of macrophages, but the gene expressions of TCR and CD3 were higher than those of ordinary macrophages (**Supplementary Figures 4A–C**). These results showed that TCR^+^ macrophages were a special group of macrophages rather than T cells.

### Potential function for TCR^+^ macrophages in HPV^+/-^ HNSCC

**Figure 5:**
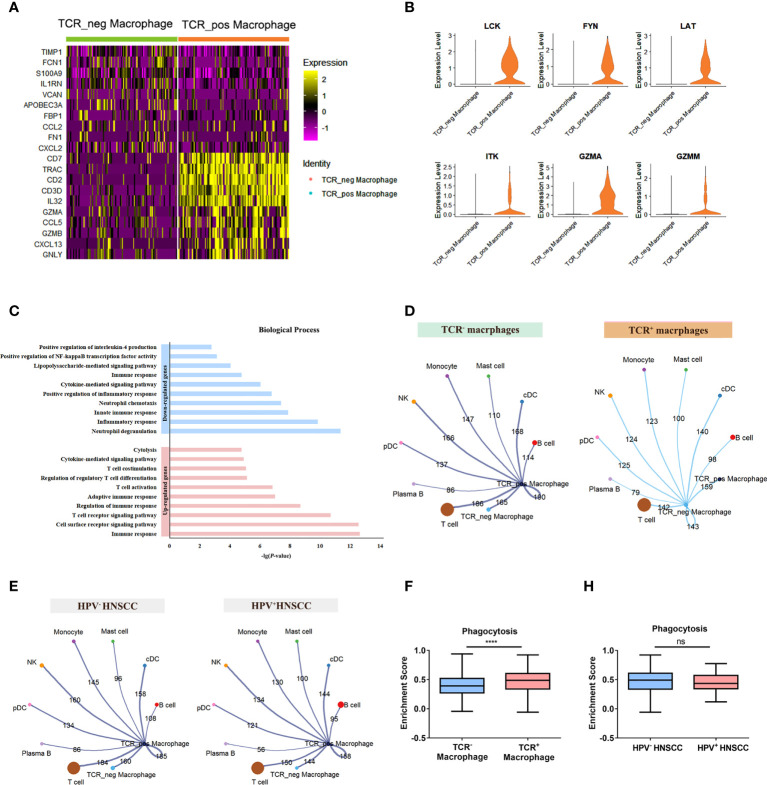
Differences between TCR^+^ macrophages and TCR^-^ macrophages. **(A)** The heatmap of the first 10 genes differentially expressed in TCR^+/-^ macrophages. **(B)** Functional enrichment of upregulated genes and downregulated genes in TCR^+^ macrophages. **(C)** UMAP plot of different genes in TCR^+^ macrophages and TCR^-^ macrophages. **(D)** The aggregated cell–cell communication analysis of TCR^+^ and TCR^-^ macrophages in HNSCC by CellChat. **(E)** The aggregated cell–cell communication analysis of TCR^+^ macrophages in HPV^+/-^ HNSCC by CellChat. **(F)** Comparisons of the phagocytosis score of TCR^+/-^ macrophages in HNSCCs ****P < 0.0001. **(H)** Comparisons of the phagocytosis score of TCR^+^ macrophages between HPV^+/-^ HNSCC patients.

To further explore the potential role of TCR macrophages in HNSCC, the differentially expressed genes between TCR^+/-^ macrophages were compared (**Figure 5A**). Among them, essential genes for TCR signaling essential genes including LCK, FYN, LAT, and ITK as well as GZMA and GZMM involved in cytotoxic effects were significantly increased in TCR^+^ macrophages (**Figure 5B**). In addition, the results of the GO analysis showed that upregulated genes of TCR^+^ macrophages were enriched in TCR signaling pathways, T-cell stimulation, activation, and differentiation pathway and cytolysis pathway, whereas the downregulated genes were enriched in positive regulation of NF-κB activity and IL-4 (**Figure 5C**). Furthermore, we evaluated the cellular interactions between TCR^+^ macrophages and other cells in HPV^+/-^ HNSCC. The results showed that TCR^+^ macrophages have differential cellular interactions with other immune cells compared with TCR^-^ macrophages (**Figure 5D**). The cellular communication network of TCR^+^ macrophages was also different in HPV^+/-^ HNSCC (**Figure 5E**). The phagocytic ability of TCR^+^ macrophages was stronger than that of TCR^-^ macrophages in HNSCC (**Figure 5F**), but there was no significant difference between HPV^+/-^ HNSCC (**Figure 5H**). However, the upregulated genes in TCR^+^ macrophages could participate in complement-related pathways in HPV^+^ HNSCC (**Supplementary Figure 1B**). The TCR^+^ macrophage was associated with the myc target v1 pathway in HPV^-^HNSCC but not in HPV^+^HNSCC (**Supplementary Figures 1C, D**). These results suggested that HPV infection influences the frequency of infiltration and specific cellular functions of TCR^+^ macrophages.

## Discussion

ScRNA-seq data provided a comprehensive resource for understanding the characteristics of macrophage subsets. A previous study has analyzed the unique states and potential plasticity of myeloid cells in HPV-related HNSCC (20). To the best of our knowledge, this is the first study providing a deep insight into the single-cell atlas of macrophage subsets and the regulation of HPV on them in HPV-related HNSCC. In addition, we identified TCR^+^ macrophages and its functional properties in HPV-related HNSCC for the first time. These results offer a valuable resource for understanding the immune landscape and immunotherapy strategies for HPV-related HNSCC.

The involvement of HPV infection in the HNSCC microenvironment always leads to a higher degree of immune response which is related to a significantly favorable prognosis (21). The infiltration percentage of the prognostic factor, macrophage, decreased significantly in HPV^+^ HNSCC based on the results of the scRNA-seq analysis in our study, while Kürten et al. also found the same phenomenon (22). Macrophages are multifunctional plastic cells and are easily modulated by factors in the microenvironment which could promote them to polarize into M1 (antitumor type) or M2 type (pro-tumor type) (23). During tumor progression, macrophages usually transform into M2 type eventually, which may be related to a favorable prognosis of HPV^+^ HNSCC (24). In breast cancer, gastric cancer, rectal cancer, and pancreatic cancer, survival analysis showed that low levels of M2 macrophage infiltration were associated with a better prognosis (23). Previous studies have shown that HPV^+^ and HPV^-^ HNSCC cell lines could induce macrophage polarization into M1 and M2 types, respectively. The ratio of M1/M2 infiltration was significantly increased in HPV^+^ HNSCC, which contributed to a favorable prognosis (8). Barbora et al. also discovered that in comparison to HPV^+^ tumors, HPV^-^ HNSCC was more infiltrated by M2 TAMs (23). HPV-promoted tumor cells secrete IL-6, thereby increasing radiosensitivity through M1 polarization of macrophages (25). Another study proved that HPV^+^ HNSCC-derived exosomal miR-9 induces macrophage polarization to the M1 type (26). In this study, we observed that macrophages in HPV^+^ HNSCC have the same dynamic change. According to the result of pseudotime analysis, the signature gene expressions of M1 macrophages in HPV^+^ HNSCC decreased more slowly, which stresses the importance of HPV’s effect on the macrophage differentiation (**Supplementary Figure 5A**). The phagocytotic ability was enhanced in HPV^+^ HNSCC, reconfirming that HPV can affect the function of macrophages. In our study, we found that lower macrophage infiltration in HPV^+^ HNSCC was positively correlated with better prognosis, but the phagocytosis score of macrophages was significantly increased in HPV^+^ HNSCC. This is possibly because most of the infiltrates around HNSCC are M2-type macrophages. The presence of HPV reduces the M2 macrophages in the tumor microenvironment and promotes the polarization of macrophages to M1 (26), so the phagocytosis of macrophages is increased. In this study, we have classified macrophages into eight populations with different genetic characteristics and functions. Most studies classify macrophages into M1 and M2, but this classification is not applicable for single-cell analysis (**Supplementary Figure 5B**). Clément et al. tried to define aortic macrophage heterogeneity into resident-like macrophages, inflammatory macrophages, and TREM2^hi^ macrophages at the single-cell level (27). Our data could find similar macrophage subsets (**Supplementary Figure 5C**), but in fact, there are still differences among the three cell subsets. Furthermore, although both C0 and C1 have the same antigen presentation function, the expression of C1QA and APOE in the C0 cell group was much lower than that in the C1 group, and the C1 group mainly belonged to monocyte-derived macrophages (**Supplementary Figure 6**). Therefore, we evaluated the resolution parameter in the analysis with Seurat and retained the original subgroup classification to facilitate a better comparison of the characteristics of macrophage subsets. However, as previously discussed, there are still some limitations in analyzing macrophages with biological information, which need algorithm optimization and experimental verification in the future.

The presence of TCR on macrophages is unconventional; however, recent studies have reported TCR expression in macrophages (9). These special macrophages also express CD3 and other molecules that are necessary for TCR signaling. In this study, the expression of essential genes for TCR signaling including LCK, FYN, LAT, and ITK was found to be significantly increased in TCR^+^ macrophages (**Figure 5B**). In addition to HNSCC, TCR^+^ macrophages have been reported to be present in tuberculous granulomas, atherosclerosis (10), and several types of cancer (colon cancer, esophageal cancer, hepatic carcinoma, triple-negative breast cancer, and melanoma) (10, 11, 28). Adriana et al. have proved that human circulating monocytes could be differentiated into CD3^+^TCR^+^ macrophages in tuberculosis (29). Zhang et al. proved that Japanese encephalitis virus infection could induce the differentiation of myeloid-derived suppressor cells into CD3^+^ macrophages in the brain (13). In this study, scRNA-seq data analysis also revealed that this population of cells may have been derived from monocytes (**Supplementary Figure 6**), but further experimental verification is needed. Several studies have shown that these unique cells could enhance phagocytosis and secrete IFN-γ, TNF, MIP-1β, and CCL2 to promote inflammation (11–13). Our results here indicated that the enhanced phagocytic ability of TCR^+^ macrophages may be critical to the prognosis of HNSCC patients. However, the inactivation of immune response in HPV^-^ HNSCC may limit the function of TCR^+^ macrophages. Therefore, despite of the increased number of TCR^+^ macrophages, the amount of TCR^+^ macrophages were not sufficient to alter the prognosis of HPV^-^ HNSCC patients. There are still a lot of more studies to be done to thoroughly understand the functions of TCR^+^CD3^+^ macrophages.

In summary, our transcriptional map of macrophages from HNSCC provided a framework for understanding the function of macrophages and revealed the dynamic nature of macrophages in HNSCC. In addition, learning the functions of TCR^+^ macrophages from many aspects will help us to understand the immune landscape in HNSCC patients and serves as an essential resource for further exploration of the roles of macrophages in HNSCC and other tumor types.

## Data availability statement

The original contributions presented in the study are included in the article/**Supplementary Material**. Further inquiries can be directed to the corresponding author.

## Ethics statement

The studies involving human participants were reviewed and approved by The Third People’s Hospital of Shenzhen. The patients/participants provided their written informed consent to participate in this study.

## Author contributions

YJ, SZ, LT, RL, JZ, SL, YP, XC, and LW contributed to the conception and design of the study. YJ, SZ, and LT performed the experiments and data analyses. YJ and SZ wrote the first draft of the manuscript. All authors contributed to the manuscript revision and read and approved the submitted version.

## Funding

The study was sponsored by the National Science Fund for Distinguished Young Scholars (No. 82102822), the Shenzhen Science and Technology Innovation Program (JCYJ20210324131607019), the Shenzhen Science and Technology Innovation Program (KCXFZ20211020163544002), and the Science and Technology Planning Projects Shenzhen Municipality (JCYJ20210324111810028).

## Acknowledgments

This research was supported by the Center for Computational Science and Engineering at Southern University of Science and Technology. We would like to thank Off-campus Practice Teaching Base of Biomedical Technology (Engineering) of Southern University of Science and Technology. We are grateful to the Cancer Hospital Chinese Academy of Medical Sciences, Shenzhen Center, for providing technical guidance and HNSCC patient tissues. We also give thanks to the Wu Lien-Teh Institute and the Key Laboratory of Preservation of Human Genetic Resources and Disease Control in Harbin Medical University for providing the experimental platform.

## Conflict of interest

The authors declare that the research was conducted in the absence of any commercial or financial relationships that could be construed as a potential conflict of interest.

## Publisher’s note

All claims expressed in this article are solely those of the authors and do not necessarily represent those of their affiliated organizations, or those of the publisher, the editors and the reviewers. Any product that may be evaluated in this article, or claim that may be made by its manufacturer, is not guaranteed or endorsed by the publisher.
