# Supplementary figures and images for: Single-cell RNA sequencing reveals TCR+ macrophages in HPV-related head and neck squamous cell carcinoma
Source: Front Immunol. 2022 Oct 27;13:1030222. doi: 10.3389/fimmu.2022.1030222 (PMC9647120; doi:10.3389/fimmu.2022.1030222)

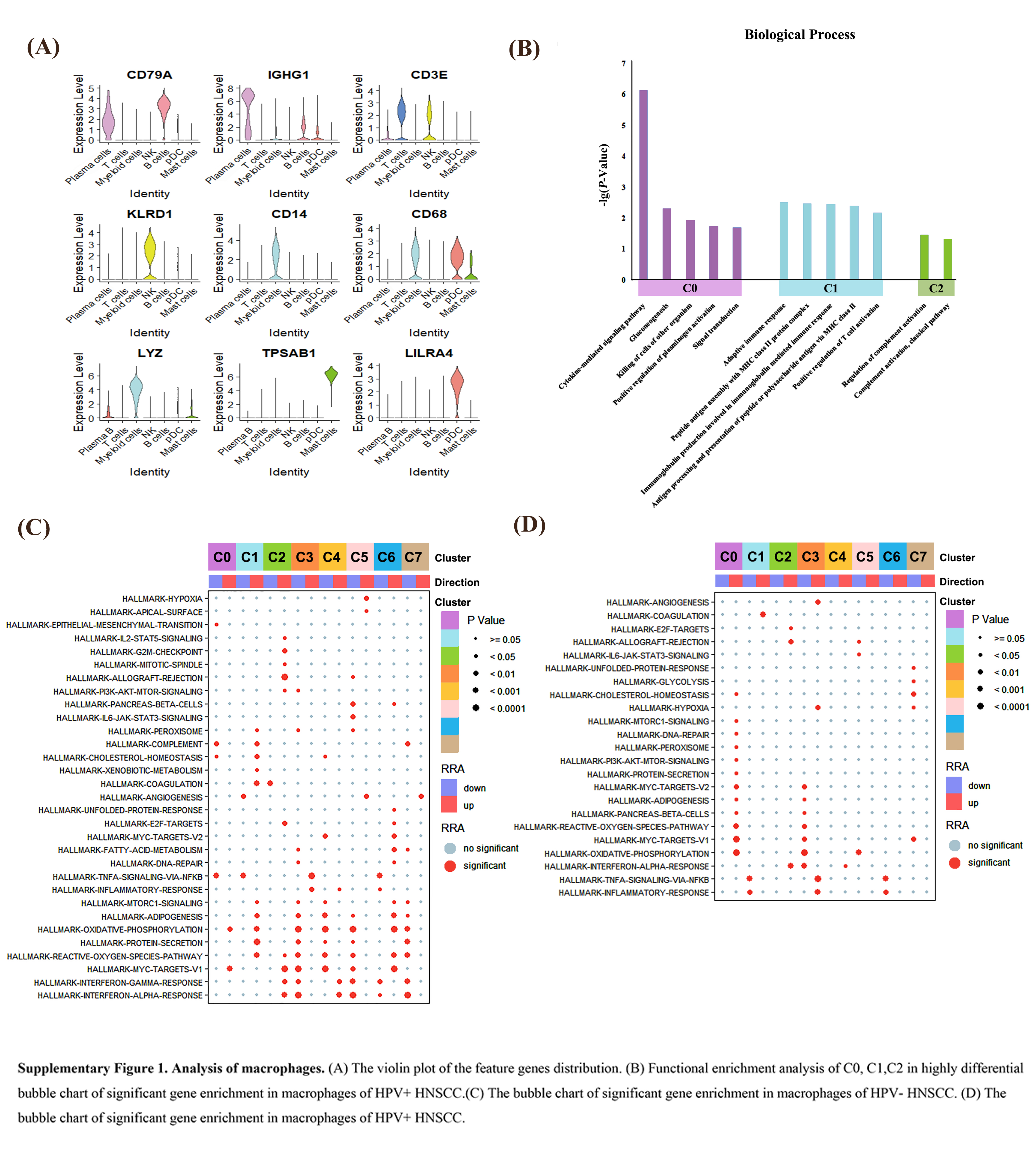

Supplement: Supplementary file 1 [file Image_1.jpeg]

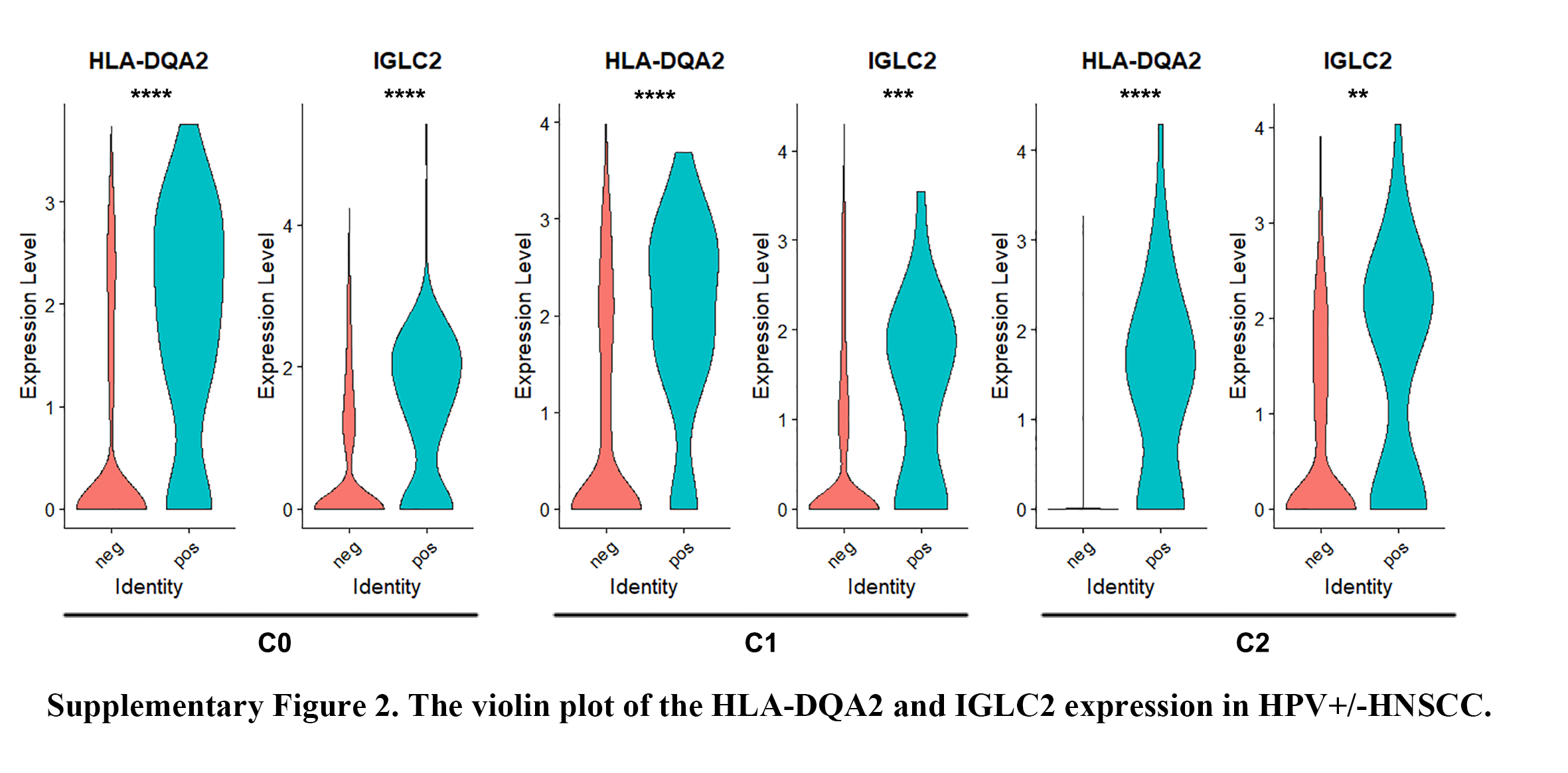

Supplement: Supplementary file 2 [file Image_2.tif]

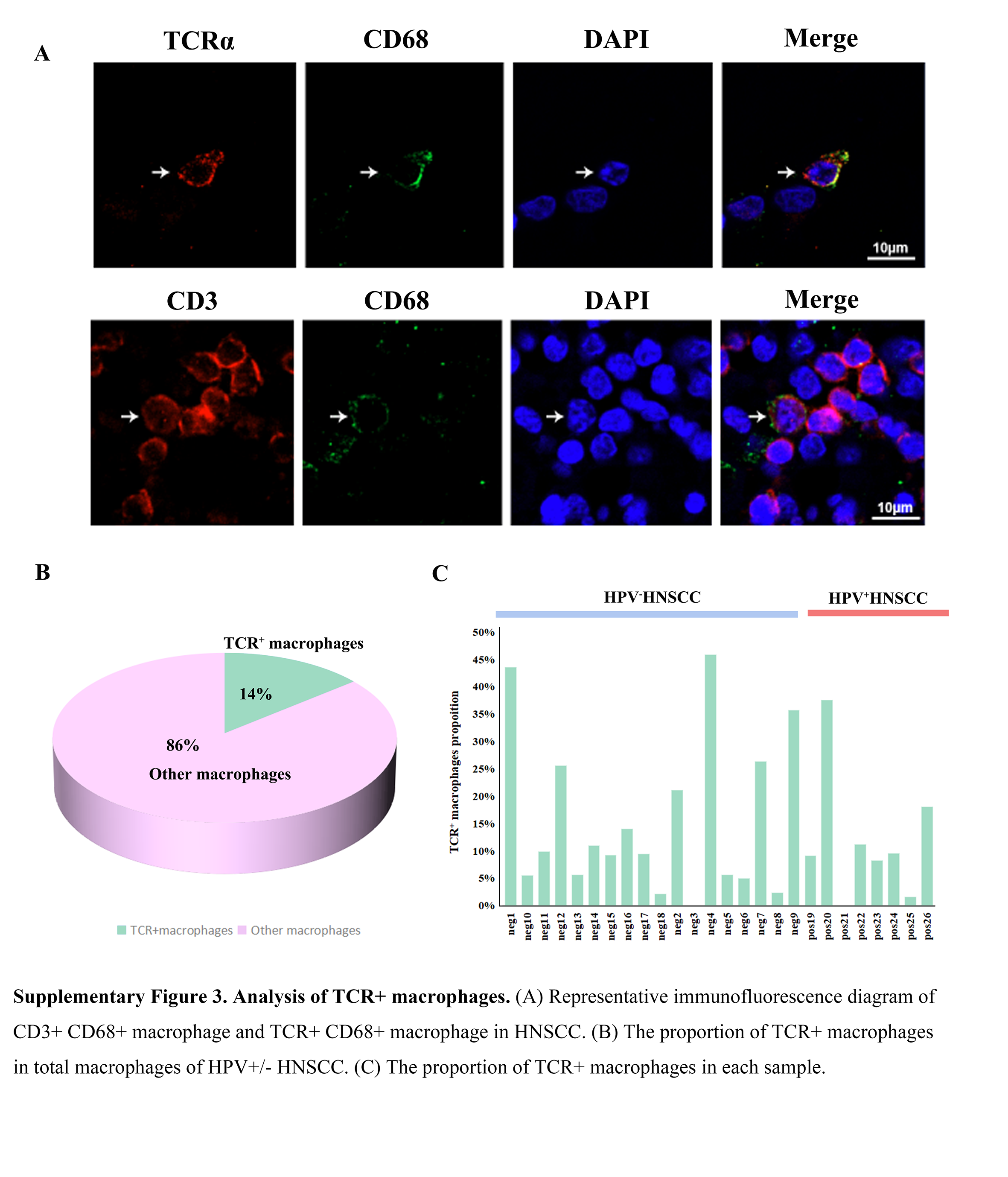

Supplement: Supplementary file 3 [file Image_3.tif]

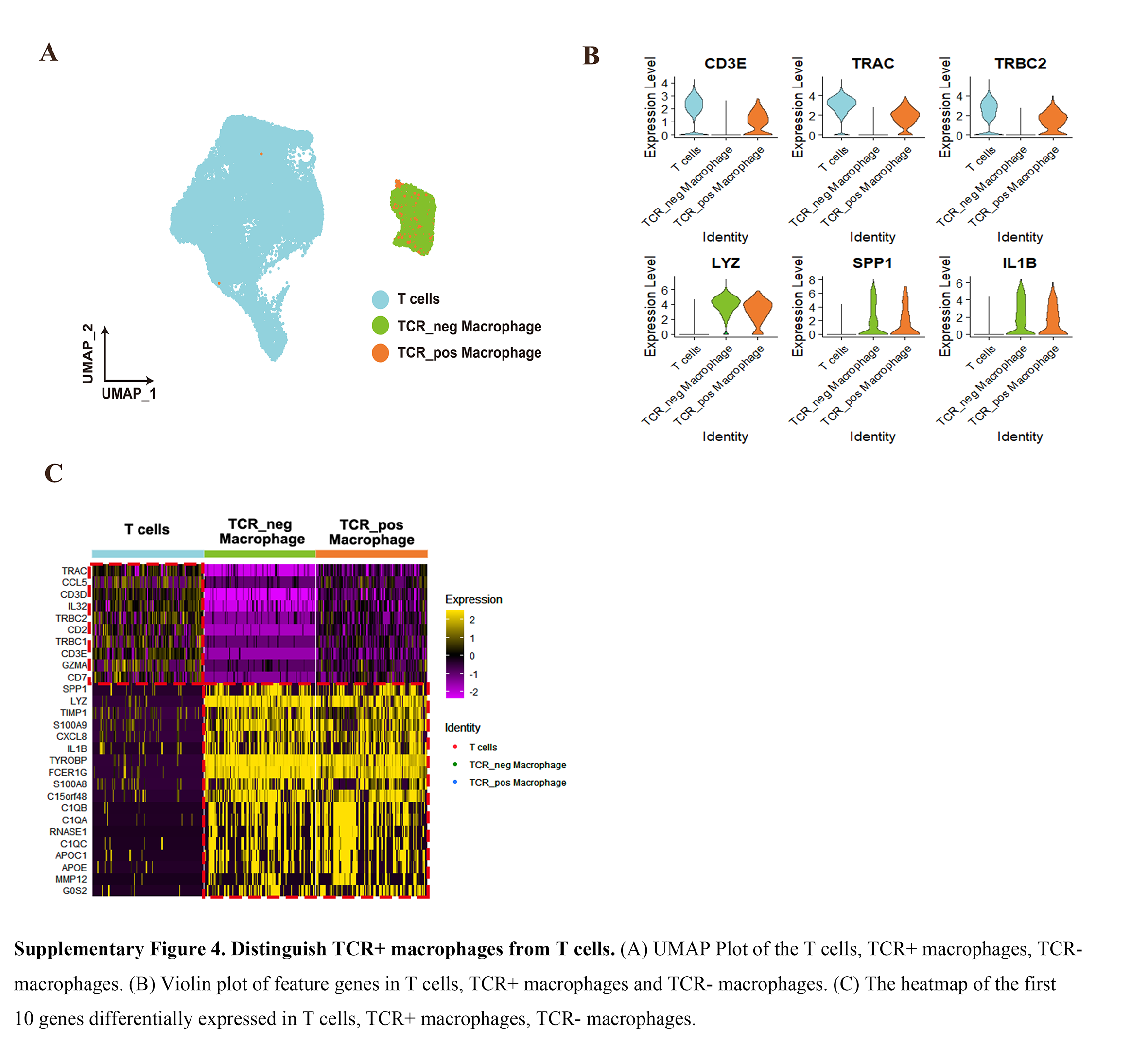

Supplement: Supplementary file 4 [file Image_4.tif]

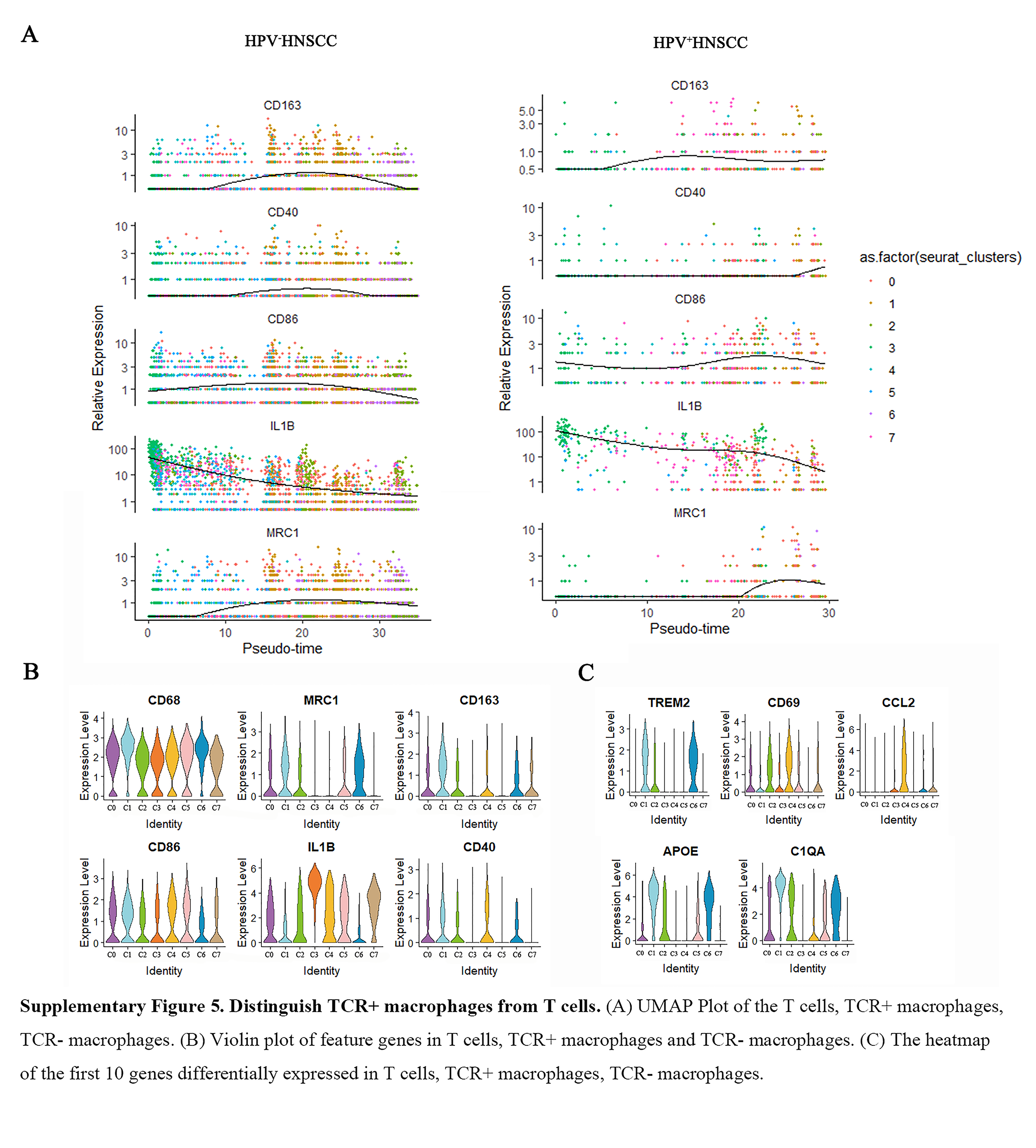

Supplement: Supplementary file 5 [file Image_5.tif]

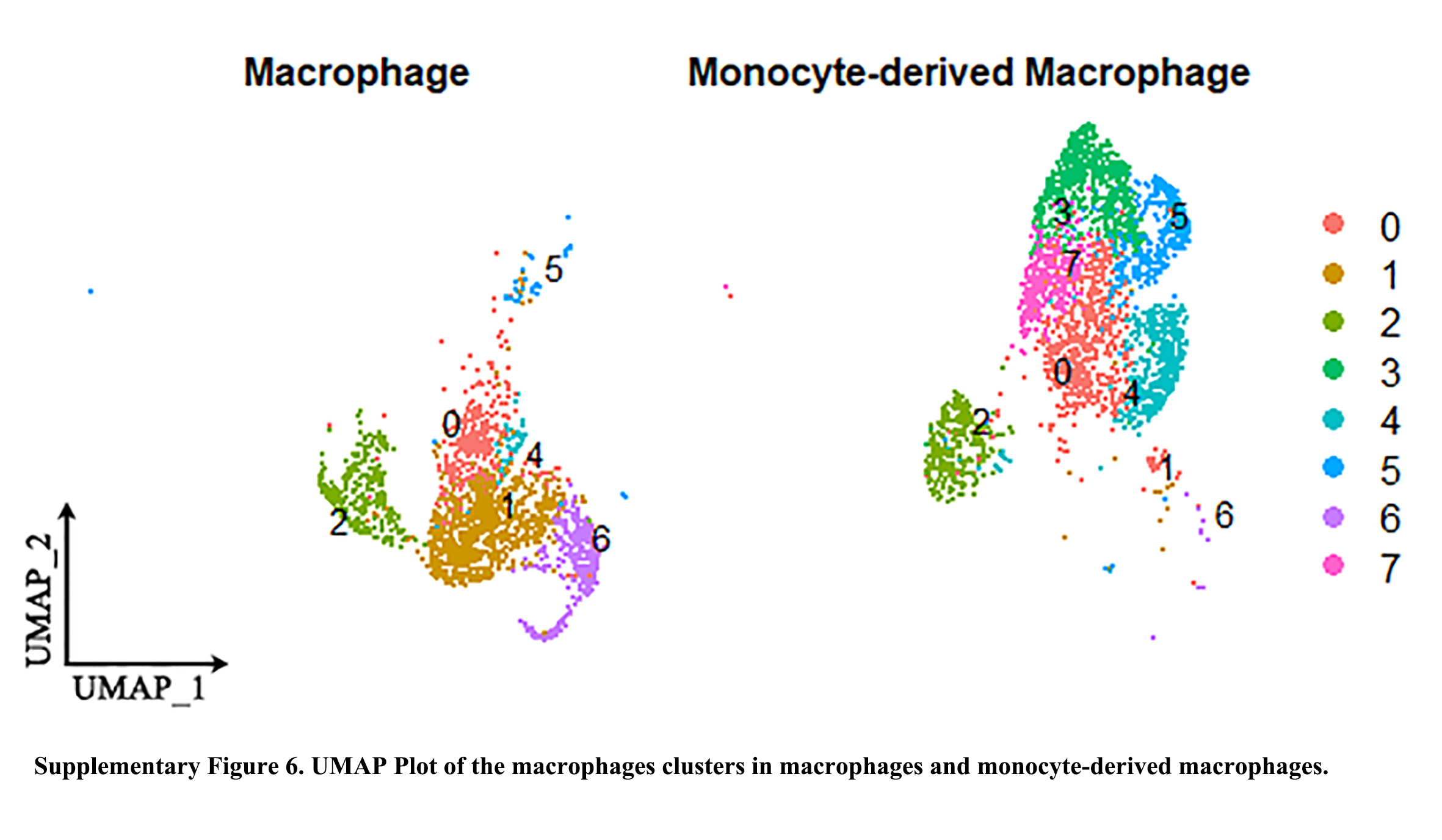

Supplement: Supplementary file 6 [file Image_6.tif]
