# Supplementary material for: Single-cell RNA sequencing reveals TCR+ macrophages in HPV-related head and neck squamous cell carcinoma
Source: Front Immunol. 2022 Oct 27;13:1030222. doi: 10.3389/fimmu.2022.1030222 (PMC9647120; doi:10.3389/fimmu.2022.1030222)
Supplement: Supplementary file 7 [file Table_1.docx]

**Supplementary Table 1**. The markers and functions of each macrophages subset

| **Cluster name** | **Representative genes** | **Unique functions** |
| --- | --- | --- |
| 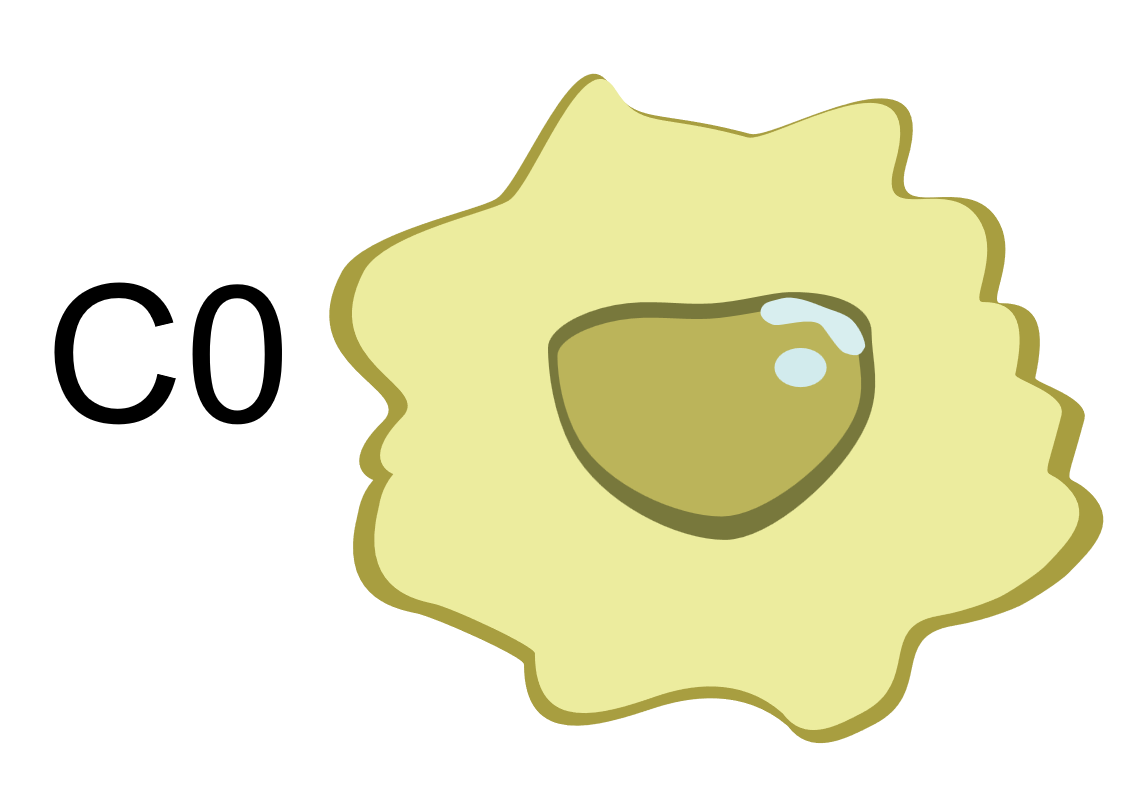 | HLA-DRB1 | Antigen processing and presentation |
|  | HLA-DMA | T cell costimulation and activicion |
|  | HLA-DQB1 | Interferon-gamma-mediated signaling pathway |
| 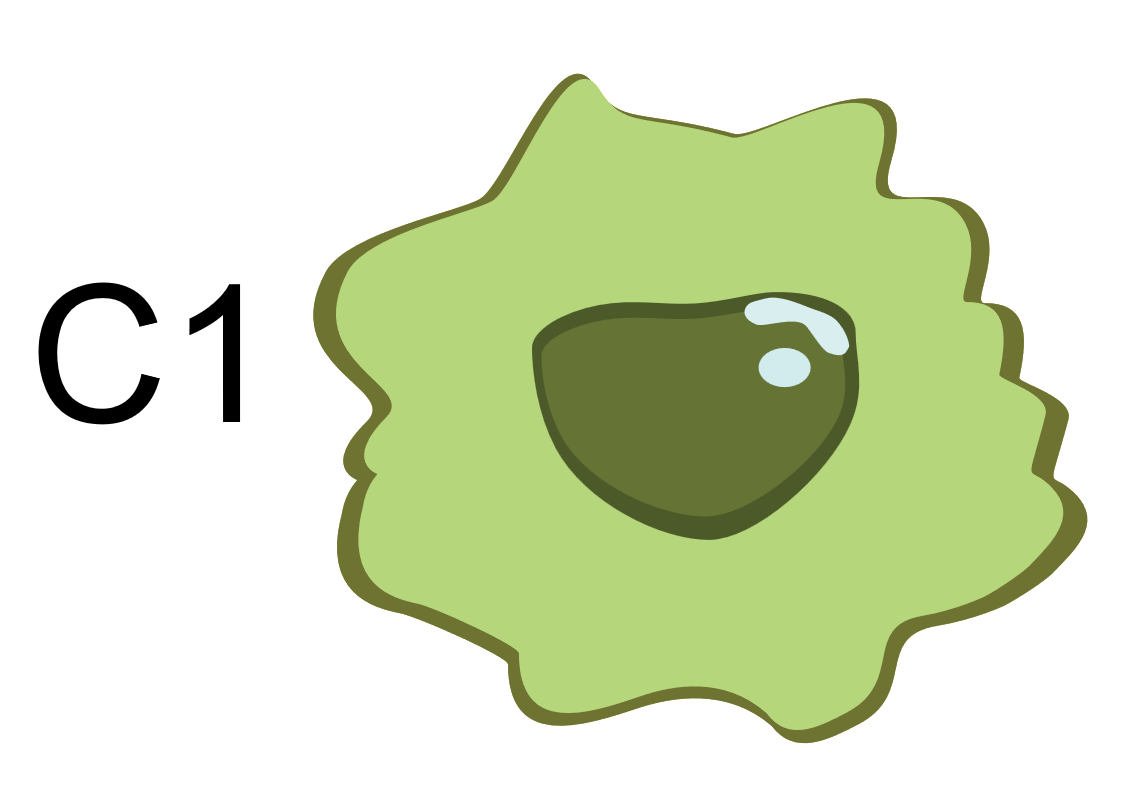 | C1QC | Antigen processing and presentation |
|  | HLA-DQA1 | T cell costimulation and activicion |
|  | HLA-DQB1 | Interferon-gamma-mediated signaling pathway |
| 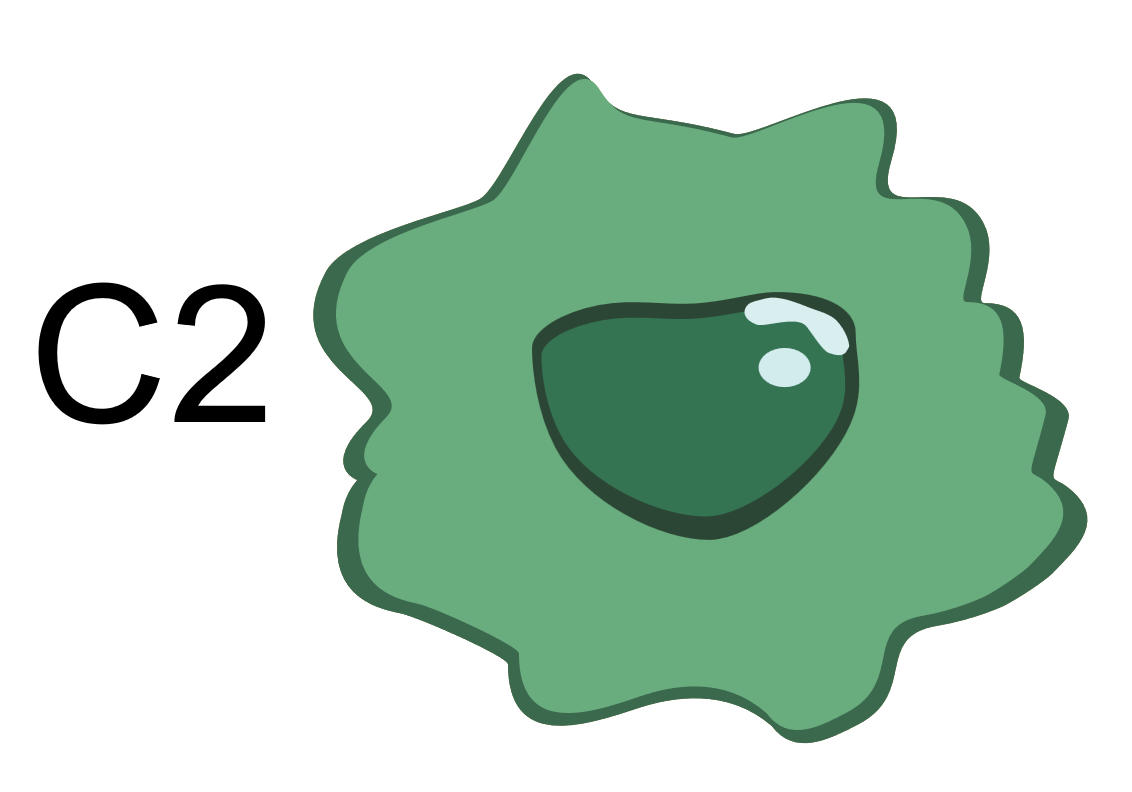 | TRAC | T cell receptor signaling pathway |
|  | TRBC2 | T cell costimulation and activicion |
|  | CD3E | Cell surface receptor signaling pathway |
| 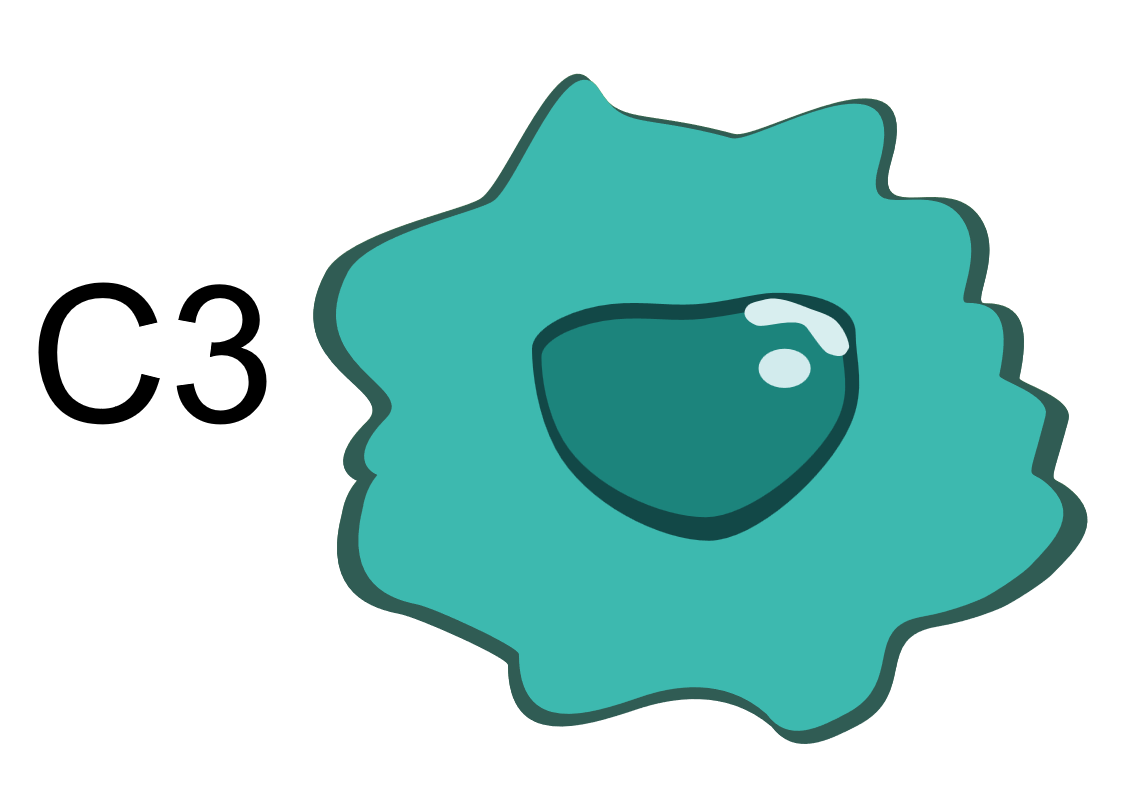 | IL6 | Inflammatory response |
|  | CXCL8 | Immune response |
|  | CCL20 | Chemokine-mediated signaling pathway |
| 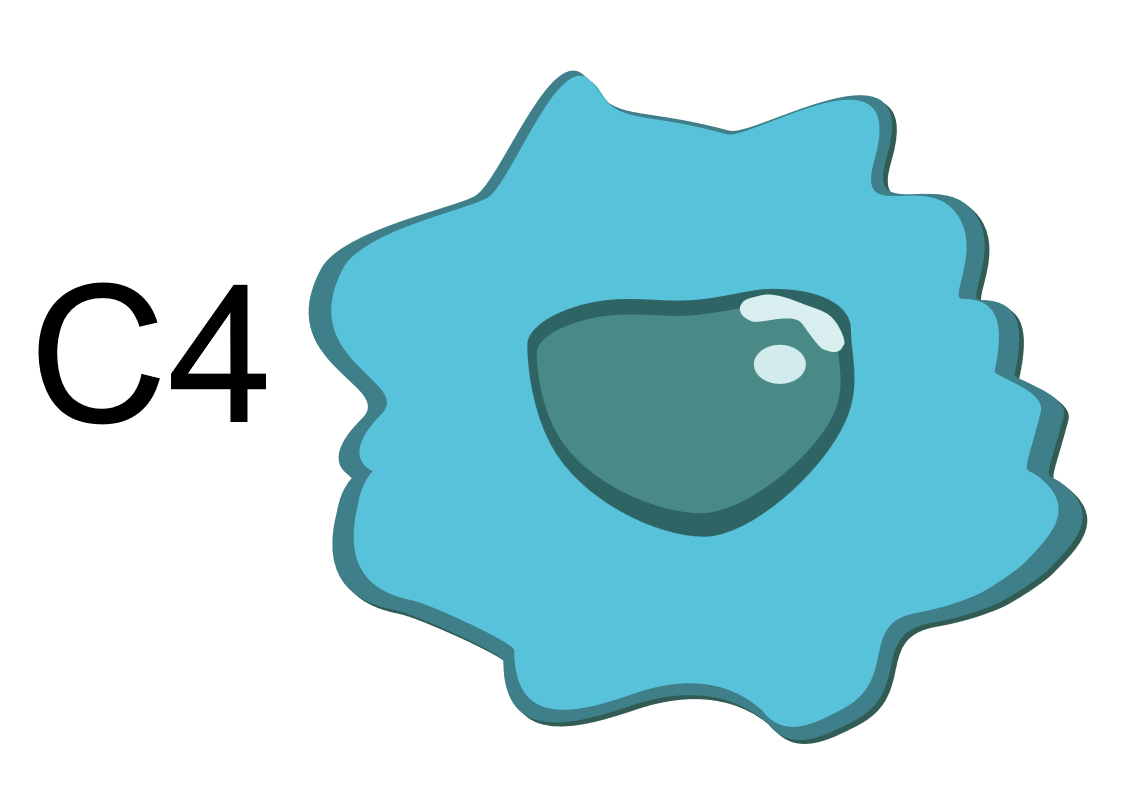 | RSAD2 | Type I interferon signaling pathway |
|  | IFIT2 | Defense response to virus |
|  | IFIT1 | Negative regulation of viral genome replication |
| 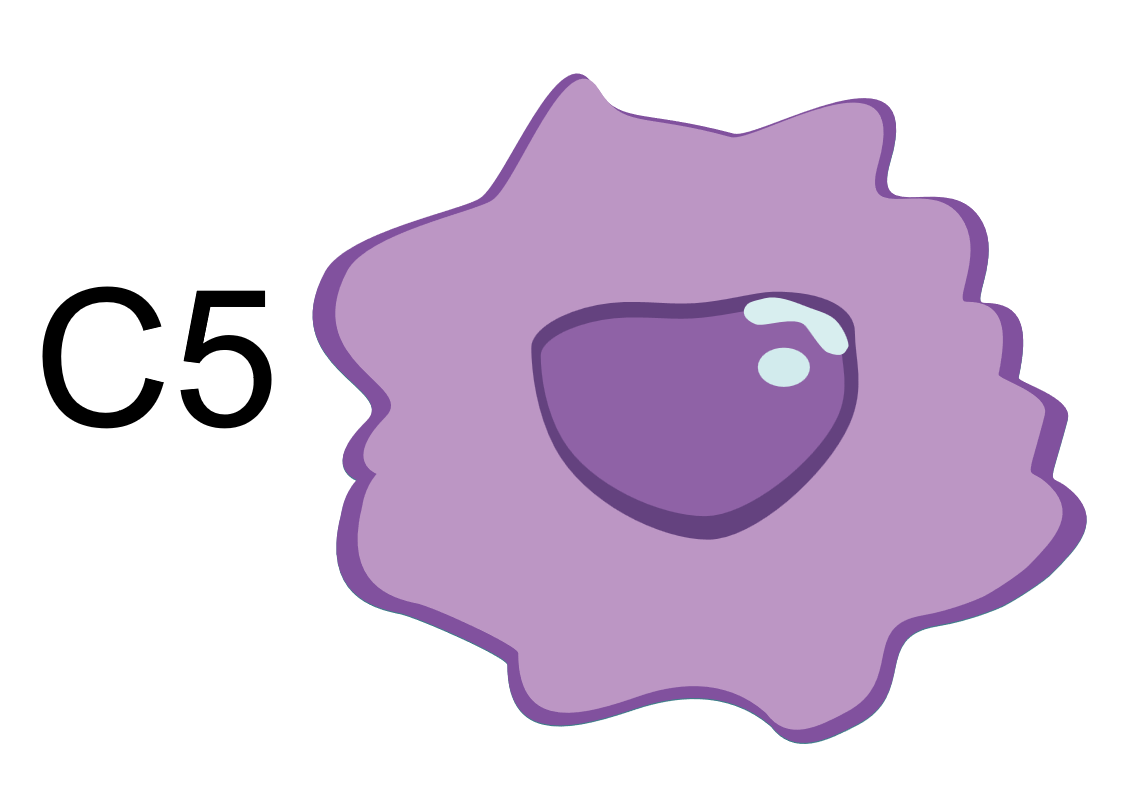 | DNAJB1 | Response to unfolded protein |
|  | HSPA1B | Regulation of cellular response to heat |
|  | BNIP3L | Regulation of apoptotic process |
| 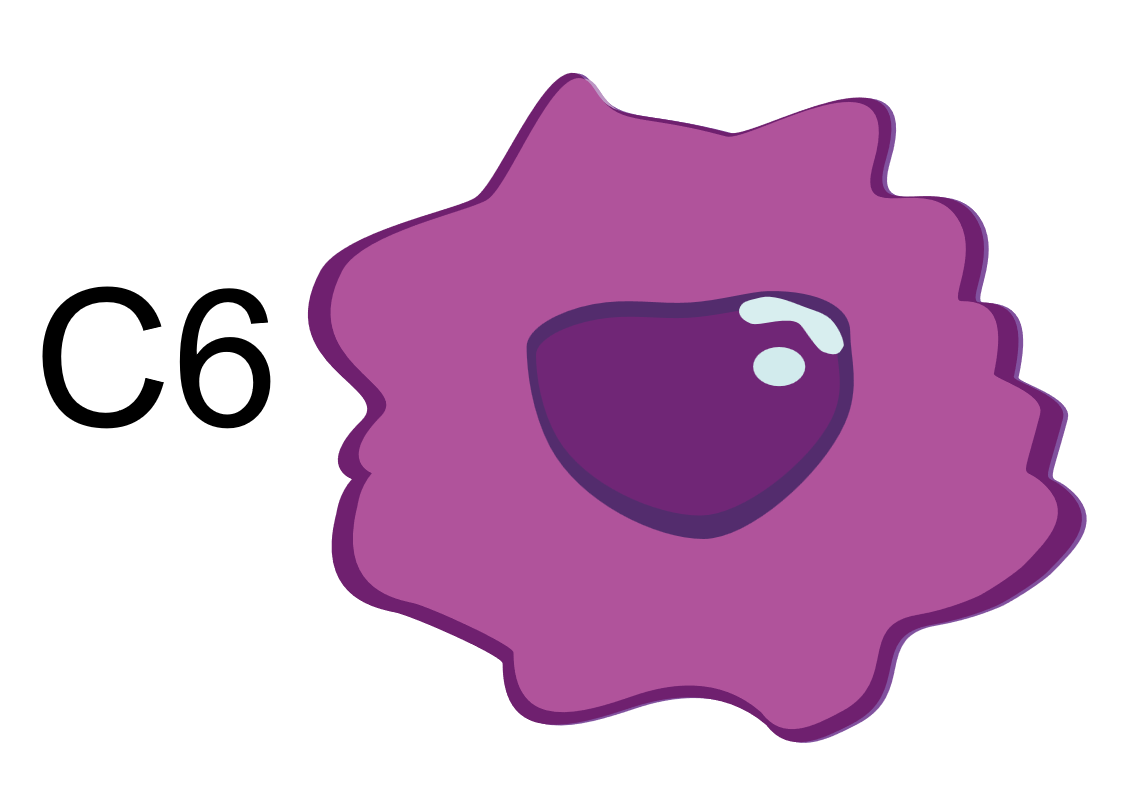 | FN1 | Triglyceride catabolic process |
|  | APOC1 | Gtriglyceride metabolic process |
|  | APOE | Lipoprotein metabolic process |
| 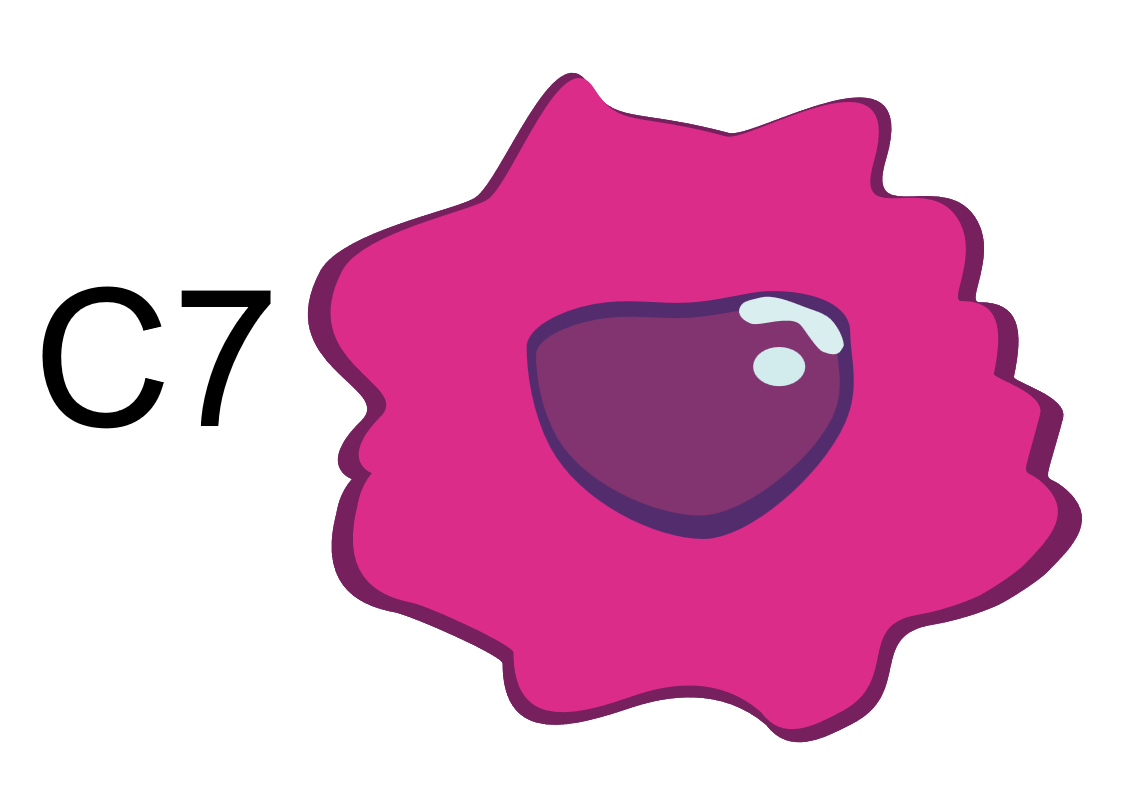 | S100A12 | Defense response to bacterium |
|  | CFP | Complement activation |
|  | IGLC2 | Positive regulation of NF-kappaB transcription factor activity |
